# A non-canonical peptide synthetase adenylates 3-methyl-2-oxovaleric acid for auriculamide biosynthesis

**DOI:** 10.3762/bjoc.12.274

**Published:** 2016-12-16

**Authors:** Daniel Braga, Dirk Hoffmeister, Markus Nett

**Affiliations:** 1 Friedrich-Schiller-Universität Jena, Department Pharmaceutical Microbiology at the Hans-Knöll-Institute, Winzerlaer Strasse 2, 07745 Jena, Germany; 2 Friedrich-Schiller-Universität Jena, Junior Research Group Synthetic Microbiology at the Hans-Knöll-Institute, Adolf-Reichwein-Strasse 23, 07745 Jena, Germany; 3 Department of Biochemical and Chemical Engineering, Technical Biology, Technical University Dortmund, Emil-Figge-Strasse 66, 44227 Dortmund, Germany

**Keywords:** adenylation, auriculamide, biosynthesis, *Herpetosiphon*, nonribosomal peptide synthetase

## Abstract

Auriculamide is the first natural product known from the predatory bacterium *Herpetosiphon aurantiacus.* It is composed of three unusual building blocks, including the non-proteinogenic amino acid 3-chloro-L-tyrosine, the α-hydroxy acid L-isoleucic acid, and a methylmalonyl-CoA-derived ethane unit. A candidate genetic locus for auriculamide biosynthesis was identified and encodes four enzymes. Among them, the non-canonical 199 kDa four-domain nonribosomal peptide synthetase, AulA, is extraordinary in that it features two consecutive adenylation domains. Here, we describe the functional characterization of the recombinantly produced AulA. The observed activation of 3-methyl-2-oxovaleric acid by the enzyme supports the hypothesis that it participates in the biosynthesis of auriculamide. An artificially truncated version of AulA that lacks the first adenylation domain activated this substrate like the full-length enzyme which shows that the first adenylation domain is dispensable. Additionally, we provide evidence that the enzyme tolerates structural variation of the substrate. α-Carbon substituents significantly affected the substrate turnover. While all tested aliphatic α-keto acids were accepted by the enzyme and minor differences in chain size and branches did not interfere with the enzymatic activity, molecules with methylene α-carbons led to low turnover. Such enzymatic plasticity is an important attribute to help in the perpetual search for novel molecules and to access a greater structural diversity by mutasynthesis.

## Findings

**Figure 1:**
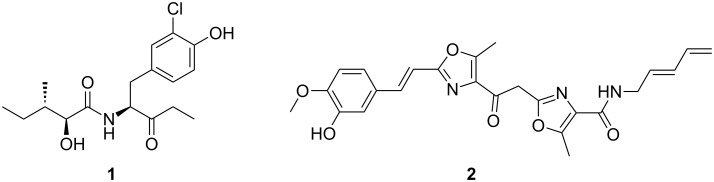
*Herpetosiphon* natural products auriculamide (**1**) and siphonazole (**2**).

**Figure 2:**
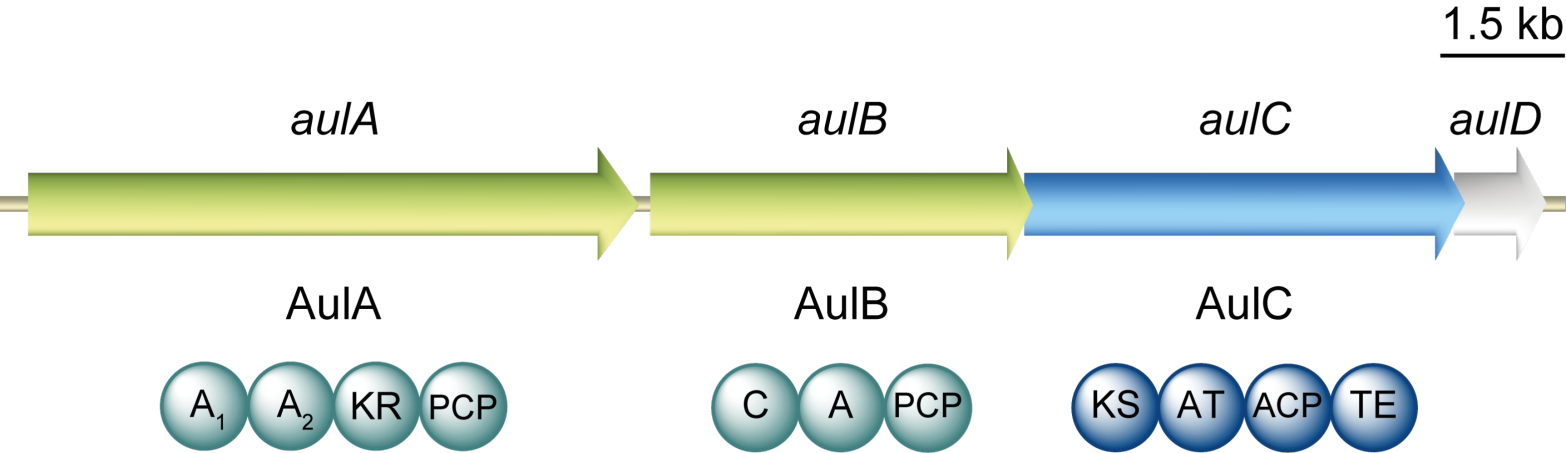
Organisation of the *aul* biosynthetic gene cluster. Circles illustrate the domain architecture of the NRPSs and the PKS present therein. Domains are abbreviated as A, adenylation; ACP, acyl carrier protein; AT, acyl transferase; C, condensation; KR, ketoreductase; KS, ketosynthase; PCP, peptidyl carrier protein; TE, thioesterase. The gene *aulD* encodes a type II thioesterase.

*Herpetosiphon aurantiacus* is a filamentous, Gram-negative bacterium with a facultative saprophytic predatory behaviour [1–2]. For a more profound insight into the predation strategies among bacteria, along with the underlying chemistry, the complete genome of *H. aurantiacus* 114-95^T^ (ATCC 23779, DSM 785) was sequenced and analysed [3]. Present as one circular chromosome and two circular plasmids, the 6.8 Mb genome of *H. aurantiacus* encodes as many as 14 biosynthesis gene clusters corresponding to 6.6% (0.45 Mb) of the genome. This capacity highlights this microorganism as a promising source of natural products. Genes for nonribosomal peptide synthetases (NRPSs) were found to be preponderant, either solely or organised in combination with polyketide synthase (PKS) genes, representing four and five clusters, respectively. Two PKS and three putative bacteriocin gene clusters complete the total set involved in the biosynthesis of natural products. Contrasting the high number of biosyntheses deduced from genomic data, knowledge on the actual natural products is limited. Recently, the dipeptide auriculamide (**1**, Figure 1), and the diterpene *O*-methylkolavelool were observed in cultures of *H. aurantiacus* 114-95^T^, providing initial evidence for the assumed secondary metabolome of this species [4–6]. Within the entire genus, **1** is only the second PKS/NRPS-derived molecule to be described, following the report on siphonazole (**2**, Figure 1) [7]. Retrobiosynthetic analysis allowed the identification of a 14,130 bp-gene cluster, now referred to as *aul*-cluster (Figure 2), which putatively encodes two NRPSs (AulA and AulB) and one PKS (AulC) possessing domains that collectively allow and plausibly explains the assembly of **1**. A gene for a type-II thioesterase is also found at the 3’ portion of the *aul* cluster that may help unload misacylated carrier protein domains [8–9].

Contrasting the standard layout of NRPSs, the amino acid sequence of one of the deduced NRPSs, termed AulA (1818 aa, 199 kDa), reveals the peculiar chimeric A_1_-A_2_-KR-PCP architecture (Figure 2) [10]. Of particular interest, the occurrence of two sequential adenylation (A) domains is a very rare feature and only preceded by PyrG from *Streptomyces pyridomyceticus* [11].

Since the lack of a genetic system for *H. aurantiacus* makes the use of reverse genetics prohibitive, we sought to provide biochemical evidence for the participation of this unusual NRPS in the biosynthesis of **1**. AulA is suggested to incorporate L-isoleucic acid (= 2-hydroxy-3-methylvaleric acid). The domain architecture indicates the substrate undergoes no other chemical modification besides a reductive step after being tethered to the PCP domain by the PKS-type ketoreductase domain (KR), as reported for other natural products, such as pyridomycin [11], cereulide, valinomycin [12], and bacillaene [13]. Hence, the molecule to be recognized and activated by AulA would be 3-methyl-2-oxovaleric acid (**3**).

Seminal work with gramicidin synthetase from *Bacillus brevis* led to the identification of ten positions within an A domain (PheA), collectively referred to as nonribosomal code [14], that control substrate selectivity. Further research started to establish a relationship between this code and structural requirements of the monomers to be recognised and incorporated to form the product [15–16]. In silico tools to identify the nonribosomal code, namely PKS/NRPS Analysis [17] and NRPSpredictor2 [18], are often accurate for the analysis of bacterial NRPSs. Yet, in our case, none retrieved any result after the analysis of AulA-A_1_. A subsequent manual inspection further revealed that the acyl-activating consensus motif is hardly conserved in AulA-A_1_. Moreover, the strictly invariant residue Asp413, which is essential for adenylate binding [14] was replaced by a tyrosine residue in AulA-A_1_. We hence concluded that this domain cannot function as an adenylating enzyme and is likely skipped during the biosynthetic assembly. Inspection of AulA-A_2_ with PKS/NRPS Analysis [17] and NRPSpredictor2 [18] yielded the nonribosomal code G-I-F-W-L-G-A-S-G-- (Table 1). Although the last position was not detected, evidences support its occupancy by a remarkably conserved lysine residue (K517 in PheA) [19], whose side chain counters the negative charge of the substrate’s carboxy group [14,20]. Also, the relationship between the expected substrate and the nonribosomal code of AulA-A_2_ posed itself as a conundrum. The first position (D235 in PheA) is normally indicative of the substrate class to be used by the NRPS. Curiously, in AulA-A_2_ this corresponds to a glycine residue, associated with the activation of anthranilic acid [21–22] and diverts from what is often observed for the activation of aliphatic or aromatic α-keto acids, where the nonribosomal code starts with a valine residue [23]. In the face of this preliminary analysis it remained elusive if and how AulA-A_1_ would contribute to the biosynthesis of auriculamide, e.g., through structural support for the catalytic role of AulA-A_2_, as noticed with a fungal A domain [24]. In order to evaluate the individual biosynthetic contribution of each A domain, we assembled two constructs to express *aulA* both as full-length gene and as an artificial open reading frame solely encoding the AulA-A_2_ domain, the α-keto reductase domain, and the terminal carrier protein. Independently, *E. coli* KRX was transformed with both constructs for heterologous production of the respective *N*-terminally hexahistidine fusion proteins, which were purified by metal affinity chromatography (Supporting Information File 1, Figure S1).

**Table 1:** Deduced nonribosomal code for *H. aurantiacus* AulA-A_2_ and the comparison with other α-keto acid activating NRPSs.

Enzyme	GenBank Accession #	Nonribosomal code										Substrate

PksJ	P40806	V	G	M	W	N	G	A	S	V	K	4-methyl-2-oxovaleric acid
CesA-A_1_	ABD14711	V	G	M	W	N	G	T	S	I	K	4-methyl-2-oxovaleric acid
CesB-A_1_	ABD14712	V	G	M	W	N	G	V	S	V	K	2-oxovaleric acid
PyrG-A_2_	AEF33080	V	G	M	T	I	G	A	S	G	K	3-methyl-2-oxovaleric acid
AulA-A_2_	ABX05055	G	I	F	W	L	G	A	S	G	K	3-methyl-2-oxovaleric acid

**Figure 3:**
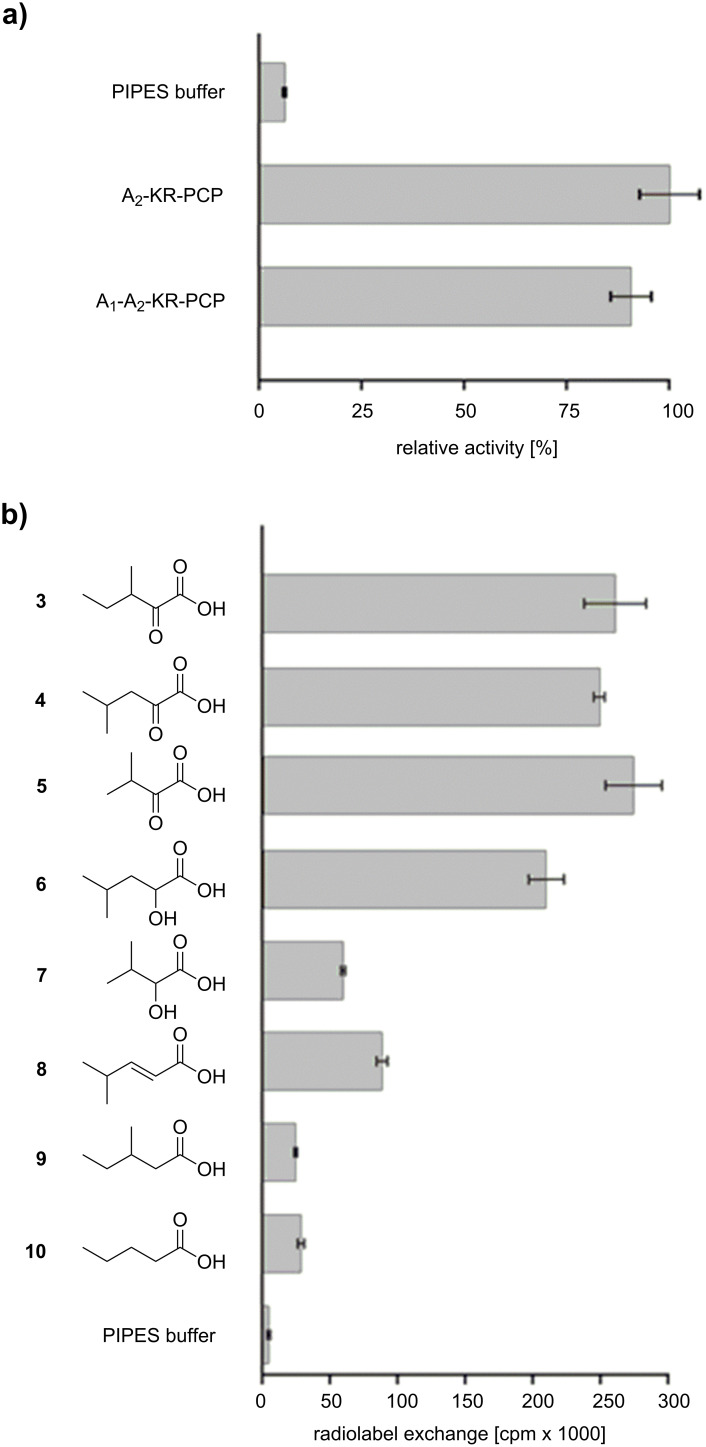
Testing of AulA (A_1_-A_2_-KR-PCP) and a truncated variant (A_2_-KR-PCP) in the ATP-[^32^P]pyrophosphate exchange assay using 3-methyl-2-oxovaleric acid as a substrate. Relative activities are referenced to the A_2_-KR-PCP enzyme (a). Substrate specificity of recombinant AulA in the ATP-[^32^P]pyrophosphate exchange assay (b). All bar diagrams depict the substrate-dependent exchange based on the arithmetic mean of triplicate reactions. Error bars represent the standard deviations. Substrates with chiral centres were tested as racemic mixtures. PIPES buffer was used as negative control.

To probe their enzymatic activity, the two purified AulA fusion proteins were subjected to the ATP-[^32^P]pyrophosphate exchange assay. In this assay, the protein is incubated with a potential substrate, ATP and radioactive pyrophosphate. The reversible back exchange of [^32^P]pyrophosphate into ATP is quantified by scintillation counting after solid phase capture of ATP on activated charcoal [25]. Both recombinant AulA variants tested against the assumed substrate **3** led to similar turnover (Figure 3a) which demonstrates that the A_1_ domain is not essential for adenylation of **3** and PCP loading. Further functional characterization was carried out using the native four-domain enzyme A_1_-A_2_-KR-PCP. Maximum turnover of **3** was observed at pH 7.0 and 30 °C. For more insight into the structural requirements of substrates, we assayed AulA against different molecules similar to **3**, varying the functional group at the α-carbon, position and number of methyl substituents, and chain length (Figure 3b, compounds **4**–**10**). As anticipated, the presence of an α-carbonyl notably influenced a successful adenylation. In the case of the tested α-keto acids, the differences in the chain size or position of the methyl group did not seem to play a role, as demonstrated by the equal enzymatic preference for **3** (261,000 cpm), **4** (249,000 cpm) and **5** (275,000 cpm). Conversely, the activation of α-hydroxy acids was not uniform. 2-Hydroxy-4-methylvaleric acid (**6**) could also be recognized by the NRPS, albeit a slightly lower radiolabel exchange (210,000 cpm) followed its incubation with the enzyme. Interestingly, the assay of 2-hydroxy-3-methylbutyric acid (**7**) resulted in a major decrease in the radiolabel exchange (60,000 cpm) when compared to its α-keto acid analogue. Molecules possessing a methylene α-carbon were not suitable substrates for AulA. 4-Methylpent-2-enoic acid (**8**) was only modestly activated (89,000 cpm), while reactions with **9** (24,000 cpm) and **10** (28,000 cpm) resulted in negligible substrate turnover.

Our biochemical in vitro results highlight AulA as apt to take part on a NRPS/PKS complex for the biosynthesis of auriculamide, given the activation of 3-methyl-2-oxovaleric acid by its second adenylation domain. Our results also contribute to hone algorithms used to predict substrates from nonribosomal codes. Moreover, we describe how this enzyme is pliant to minor structural variations of that molecule, enabling future attempts to generate auriculamide analogues as potential new drug candidates.

## Supporting Information

File 1 Complete experimental details.
