# Supplementary material for: A non-canonical peptide synthetase adenylates 3-methyl-2-oxovaleric acid for auriculamide biosynthesis
Source: Beilstein J Org Chem. 2016 Dec 16;12:2766–70. doi: 10.3762/bjoc.12.274 (PMC5238615; doi:10.3762/bjoc.12.274)
Supplement: File 1 — Complete experimental details. [file Beilstein_J_Org_Chem-12-2766-s001.pdf]

Supporting Information File

for

A non-canonical peptide synthetase adenylates 3-methyl-2-oxovaleric acid for auriculamide biosynthesis

Daniel Braga[1,2], Dirk Hoffmeister[1] and Markus Nett*[3]

Address: [1]Friedrich-Schiller-Universität Jena, Department Pharmaceutical Microbiology at the Hans-Knöll-Institute, Winzerlaer Strasse 2, 07745 Jena, Germany, [2]Friedrich-Schiller-Universität Jena, Junior Research Group Synthetic Microbiology at the Hans-Knöll-Institute, Adolf-Reichwein-Strasse 23, 07745 Jena, Germany and [3]Department of Biochemical and Chemical Engineering, Technical Biology, Technical University Dortmund, Emil-Figge-Strasse 66, 44227 Dortmund, Germany

Email: Markus Nett - markus.nett@bci.tu-dortmund.de

*Corresponding author

Complete experimental details

Experimental details

Bacterial strains, culture conditions, and general methods ………………………. S3

Bioinformatics ………….……………………………...………………………... S3

Generation of hexahistidine-tagged constructs ……………………….………. S3

Production and purification of the recombinant proteins S4

Figure S1. SDS-PAGE of the purified recombinant AulA variants ……………… S5

Protein sequences (without the hexahistidine tag) ………………………………. S6

In vitro ATP-[^{32}P]pyrophosphate exchange assay S8

References ……………………………………………………………………….. S8

General methods

Bacterial strains, culture conditions, and general methods. *E. coli* was routinely grown at 37 °C on lysogenic broth (tryptone, 10 g/L; yeast extract, 5 g/L; NaCl, 10 g/L) amended with kanamycin (100 µg/mL) when appropriate. Strain XL-1 blue was used for cloning and preparation of plasmid DNA, whereas strain KRX was used in the heterologous production of the recombinant NRPS. Standard molecular biology procedures followed described protocols [1]. DNA purification, restriction, and ligation were performed in accordance with the instructions of the manufacturers. Kits and enzymes were purchased from NEB, Promega, Thermo Fisher Scientific, and Zymo Research. Eurofins Genomics performed the DNA sequence verification. Chemicals were acquired from Sigma-Aldrich, BD, VWR, and Carl Roth. [^{32}P]pyrophosphate was purchased from PerkinElmer.

Bioinformatics. In silico analyses of the nonribosomal code and domain disposition were performed with NRPSpredictor2 [2] and PKS/NRPS [3]. Amino acid sequences of the adenylation domains pertaining to AulA (ABX05054.1), CesA (ABD14711.1), CesB (ABK00633.1), PksJ (P40806.3), and PyrG (AEF33080.1) were aligned using ClustalW algorithm by Geneious v.9.1.5 (Biomatters, New Zealand) in order to verify the presence of a lysine residue in the last position of AulA nonribosomal code.

Generation of hexahistidine-tagged constructs. Genomic DNA of *H. aurantiacus* 114-95^{T} [4] served as template to amplify *aulA*. The amplification of the sequence encoding the full-length protein (A_1-A_2-KR-PCP) was performed with the oligonucleotide primers oDB88/89 (5’-GCT CCG GCG GAT CCA TGG AAA TGG ATT TTC AGA GT-3’ and 5’-GCA TCG CTG TCG ACT TAC CCT AAT CCT GCT TCA AG-3’ respectively). The sequence encoding the truncated protein (A_2-KR-T)

was amplified with oligonucleotides oDB89/90 (5’-CCA TAG AAT TCA TGT TGT CAA CTC AGG CCA AGC-3’). 20 µL-reactions consisted of 0.2 mM (each) dNTP, 20 pmol (each) oligonucleotide primer, 2 mM $MgCl_2$, and 0.4 U Phusion® High Fidelity DNA polymerase in the supplied buffer, and 100 ng genomic DNA as template. Thermal cycling parameters were: 30 s at 98 °C; 33 cycles of 98 °C for 10 s, 55 °C for 15 s and 72 °C for 150 s, followed by a terminal hold 5 min at 72 °C. Amplicons of full and partial *aulA* were ligated into pET28a(+) to generate pDB039 (10,813 bp) and pDB042 (9,181 bp), respectively. The resulting plasmids encode the respective *N*-terminal hexahistidine fusions.

Production and purification of the recombinant protein. For heterologous production of the recombinant AulA variants, *E. coli* KRX (Promega) was used as host and grown in 400 mL LB medium supplemented with 100 µg/mL kanamycin. After reaching an OD_{600} = 0.7, gene expression was induced by adding 0.1% (w/v) L-rhamnose, followed by further cultivation at 16 °C for 18 hours. Cells were harvested by centrifugation (3,200*g*, 50 min, 4 °C) and suspended in lysis buffer (20 mM imidazole, 50 mM sodium phosphate, 300 mM sodium chloride, pH 8.0). Cells were disrupted with a Sonopuls ultrasonic sonifier (Bandelin). The lysate was centrifuged (17,000*g*, 30 min, 4 °C) to remove cellular debris. The proteins were purified by metal affinity chromatography using an Äkta FPLC (GE Healthcare) equipped with a HisTrap HP column (GE Healthcare), subjecting the sample to a stepwise imidazole gradient (20–500 mM) in lysis buffer. Proteins were desalted on a PD-10 column (GE Healthcare) and eluted with reaction buffer (100 mM PIPES, pH 7.0, 5 mM $MgCl_2$, 125 nM EDTA). Purification was confirmed by SDS-PAGE.

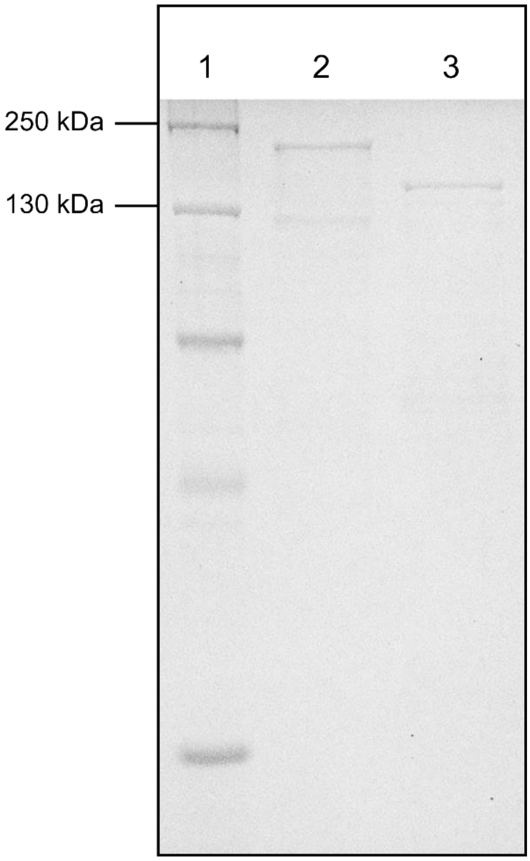

Figure S1: SDS-Polyacrylamide gel of the purified recombinant AulA. The electrophoretic run was performed in 12% Laemmli gels at 35 mA.

(1) Protein ladder

(2) AulA (A_1-A_2-KR-PCP, 1852 aa, 203.1 kDa)

(3) Truncated AulA (A_2-KR-PCP, 1308 aa, 143.8 kDa).

Protein sequences (without the hexahistidine tag)

AulA (A_1-A_2-KR-T)

MEMDFQSIQRRFATAVEQRSSQAALRYHDQVVSYHELAEHAQRIASGLANQQVGV

NTNVAIQLTNPIDVCSTILATLLLGARYALLSPNLAKLRLQQVLARQQFVLVGSAASN

NLAANYIEFEQLANSELAEITPHSATAESLIGLSLASNPSGLIEAGQLSQTNLLSFIDFN

LSKAKVSFQQSLWLGEEFNDFSAFASLATLASGGTLRFSTLETLDHDLDEQAQTLM

LTLATLGQLFAQQSALPKVRHILSSGEGLLDGEALKQQLKQQQTAWHNYYGFPAFQ

LLTVVGANTQTQTASQIHSGKPVPHTQALILDQHKQLAPIGLTGELYVAGAGVFAGF

EQAQLNAERFIASPFAADTQLYQTRYLARWQDDGRLSISGSLDSTIELAATPILLQEV

ERLLEQHPAIVECCIVRRITLSNTEQLTGFVVAKQRVEPSEILNYLEDQLDCQLANLG

LIQVDQLPRTADGQLDRQQLAQTNLLDNRQIADLQAQLQQAANGAELAVVAQPILPV

SSPLHIDDLVPMVETSNFGTSQRTISEQPVEMLSTQAKPALAVGPPLIKAEQAPLTLA

EALVLAAKHYPEHGISYIEADGKALFQSYAALLADAEAVLAGLRAAGLKHGQHVVLQ

FAHNEPFVVAFWACMLGGFTAVPLALPNSSDPNNPAVSKLYNTWQTLEQPLIVSEQ

ASFSLLQRIFNGLGVVKPAIQITEQLRQHQPDQQHQHLAPQDSALLLFTSGSTGLPK

GVELSHHNIISRSKASAQHNRFDHNDVSLNWMPLDHVGGIVMFHVHDVCLGCRQIQ

AKTDYILEDPVRWLDLLEQYRATITWSPNFAYALINDQHERVNSRRRNLSSLRFILNG

GEGINKQTALNFLGLLQAHGLPATAMHPAYGMSETSSGISSSDQLVLGATTGFHEL

DQASLTGVIQPASADSIGVAFVEVGAPLPGVSLRIVNTNNQLLSEDLIGRLQIQGPTIT

AGYYRNPELNREVFTDDGWFTTGDLAFLHQGRLTIAGREKDVIIINGINYHNHEIEAL

VETIEGVEVSYTAACSVPSKHTGGTESLVIFYVSKSAEFDQQLAQINQIREVVVQKIGI

NPSYVLPVAKSDIPKTAIGKIQRSQLSQRFINGEFSSITKPIDLALANQQTLPRWFFSK

QWQPVSKRHNSALLKPSYAIFSDDTTLARELIDVLEQHHRDWVLISAGETFSQQGQ

HYTINLHDPEHYHQIAATLAATNIHDYVHLYSCDLPSEIEHVGDLAAAQYRGTYSLLF

LTQALAKQKLSQASLTVVSQRSHAINQSDQVIYAAAPIHGLLKTMPLEIDWLSCQHV

DLDAASATTNSQQIYYELAQPKPSAEVAYRAGQRLVPQLVEAEMAQSSPVESPLVK

GGLYLVTGGLGGIGSQFARWLLQNYNARLLITGSTELPLGSDWAKHLGTDSSLSKR
LRAYKDLIDISNDVHYQAVDITDSAQLARLINDAEQRWNQPLAGVFHFAGAGNLAYH
WTVMDHHWITNESLATFEMMFAPKVYGTWALQRALSQRPELPIVAMSSINSFFGGA
TFSAYSAANSFLDSFMLHQRQTSHPKALCLNWTQWDNIGMSLNNPQQIRSLSAER
GYNVIGLQQGLQSLLAGISQNQYPLLMIGLNADSPALRQHLAVSQPLQQRINLYTTH
QHGPLSHDRYRQLANSYFGSATLEWYRVAELPRTSSGAIDLAALGQLDATNQQTAL
DQPTNIIEEQLVSIWQEILGKPKIGIHDNFFALGGHSLLATQLVSRLRDGFNLEVRLYQ
LFAAPTIAELANCIAELQLEQIDSAEMDALLAELEGLSEAELEAGLG

Partial AulA (A_2-KR-T)

MLSTQAKPALAVGPPLIKAEQAPLTLAEALVLAAKHYPEHGISYIEADGKALFQSYAA
LLADAEAVLAGLRAAGLKHGQHVVLQFAHNEPFVVAFWACMLGGFTAVPLALPNSS
DPNNPAVSKLYNTWQTLEQPLIVSEQASFSLLQRIFNGLGVVKPAIQITEQLRQHQP
DQQHQHLAPQDSALLLFTSGSTGLPKGVELSHHNIISRSKASAQHNRFDHNDVSLN
WMPLDHVGGIVMFHVHDVCLGCRQIQAKTDYILEDPVRWLDLLEQYRATITWSPNF
AYALINDQHERVNSRRRNLSSLRFILNGGEGINKQTALNFLGLLQAHGLPATAMHPA
YGMSETSSGISSSDQLVLGATTGFHELDQASLTGVIQPASADSIGVAFVEVGAPLPG
VSLRIVNTNNQLLSEDLIGRLQIQGPTITAGYYRNPELNREVFTDDGWFTTGDLAFLH
QGRLTIAGREKDVIIINGINYHNHEIEALVETIEGVEVSYTAACSVPSKHTGGTESLVIF
YVSKSAEFDQQLAQINQIREVVVQKIGINPSYVLPVAKSDIPKTAIGKIQRSQLSQRFI
NGEFSSITKPIDLALANQQTLPRWFFSKQWQPVSKRHNSALLKPSYAIFSDDTTLAR
ELIDVLEQHHRDWVLISAGETFSQQGQHYTINLHDPEHYHQIAATLAATNIHDYVHLY
SCDLPSEIEHVGDLAAAQYRGTYSLLFLTQALAKQKLSQASLTVVSQRSHAINQSDQ
VIYAAAPIHGLLKTMPLEIDWLSCQHVDLDAASATTNSQQIYYELAQPKPSAEVAYRA
GQRLVPQLVEAEMAQSSPVESPLVKGGLYLVTGGLGGIGSQFARWLLQNYNARLLI
TGSTELPLGSDWAKHLGTDSSLSKRLRAYKDLIDISNDVHYQAVDITDSAQLARLIND

AEQRWNQPLAGVFHFAGAGNLAYHWTVMDHHWITNESLATFEMMFAPKVYGTWA
LQRALSQRPELPIVAMSSINSFFGGATFSAYSAANSFLDSFMLHQRQTSHPKALCLN
WTQWDNIGMSLNNPQQIRSLSAERGYNVIGLQQGLQSLLAGISQNQYPLLMIGLNA
DSPALRQHLAVSQPLQQRINLYTTHQHGPLSHDRYRQLANSYFGSATLEWYRVAEL
PRTSSGAIDLAALGQLDATNQQTALDQPTNIIEEQLVSIWQEILGKPKIGIHDNFFALG
GHSLLATQLVSRLRDGFNLEVRLYQLFAAPTIAELANCIAELQLEQIDSAEMDALLAE
LEGLSEAELEAGLG

In vitro ATP-[^{32}P]pyrophosphate exchange assay. All reactions were run in triplicates at 30 °C. The reactions were composed of reaction buffer (described above), 1 mM individual substrates, 100 nM purified enzyme and 0.1 µM [^{32}P]pyrophosphate (50 Ci/mmol). The reaction volume was 100 µL. The reactions were stopped after 30 min and further processed as described [5]. To establish optimum conditions for enzymatic activity, 3-methyl-2-oxovaleric acid served as a substrate in reactions carried out at varied temperatures (4–56 °C) and pH values (6.0–9.0) using PIPES- and TRIS-buffers. Radioisotope exchange was quantified on a PerkinElmer TriCarb 2910TR scintillation counter.

References

1. Sambrook, J.; Russell, D. W., *Molecular Cloning: A Laboratory Manual*. 3 ed.; Cold Spring Harbor Laboratory Press: 2001.

2. Röttig, M.; Medema, M. H.; Blin, K.; Weber, T.; Rausch, C.; Kohlbacher, O., *Nucleic Acids Res* **2011,** *39* (Web Server issue), W362-7.

3. Bachmann, B. O.; Ravel, J., *Methods Enzymol* **2009,** *458*, 181-217.

4. Schieferdecker, S.; Domin, N.; Hoffmeier, C.; Bryant, D. A.; Roth, M.; Nett, M., *Eur J Org Chem* **2015,** 3057-3062.

5. Schneider, P.; Weber, M.; Rosenberger, K.; Hoffmeister, D., *Chem Biol* **2007,** *14* (6), 635-44.
